# Outcome after surgery for acute right-sided colonic ischemia without feasible vascular intervention: a single center experience of 58 patients over 6 years

**DOI:** 10.1186/s12893-015-0018-0

**Published:** 2015-03-21

**Authors:** Samuel A Käser, Tara C Müller, Anna Guggemos, Ulrich Nitsche, Christoph Späth, Christoph A Maurer, Klaus-Peter Janssen, Jörg Kleeff, Helmut Friess, Dirk Wilhelm, Franz G Bader

**Affiliations:** Department of Surgery, Klinikum rechts der Isar, Technische Universität München, Ismaningerstrasse 22, 81675 Munich, Germany; Hirslanden Private Clinic Group, Beausite, Schänzlihalde 11, CH-3000 Bern, Switzerland

**Keywords:** Non-occlusive mesenteric ischemia, Urgent surgery, Critically ill

## Abstract

**Background:**

The predilection site of non-occlusive mesenteric ischemia is the right-sided colon. Surgical exploration followed by segmental bowel resection and primary anastomosis or ileostomy is recommended, if vascular interventions are not feasible and conservative treatment fails. We assessed the outcome of patients in this life-threatening condition.

**Methods:**

From a prospective database 58 patients with urgent surgery for acute right-sided colonic ischemia without feasible vascular intervention (as a surrogate for non-occlusive mesenteric ischemia) were identified. Retrospectively the patients’ characteristics, reason for ischemia, extent of resection, rate of ileostomy creation, 30 day and one year mortality, and rate of ileostomy-reversal at one year postoperative were assessed.

**Results:**

Radiologically mesenteric arteriosclerotic disease was present in 54% of the patients. Vaso-occlusive mesenteric disease was suspected in 15% of the patients, but not confirmed intra-operatively. Ten patients underwent (extended) right-sided hemicolectomy with primary anastomosis (30-days mortality 20%, 1-year mortality 30%). Sixteen patients had (extended) right-sided hemicolectomy with creation of an ileostomy (30-days mortality 44%, 1-year mortality 86%, ostomy reversal in one patient). Twenty-five patients had (sub-) total colectomy with ileostomy creation (30-days mortality 60%, 1-year mortality 72%, ostomy reversal in two patients). Seven patients had exploration only (30-days mortality 86%, 1-year mortality 86%). Overall, the 30-days mortality-rate was 52% and the 1-year mortality-rate was 70%. Only 7% of the patients requiring an ostomy experienced ostomy-reversal.

**Conclusions:**

Patients with urgent surgery for acute right-sided colonic ischemia without feasible vascular intervention have a very high short and long-term mortality. The rate of ostomy-reversal is very low.

## Background

Non-occlusive mesenterial ischemia (NOMI) is a rare but life-threatening condition caused by severe hypotension in the critically ill. It often occurs after myocardial infarction, after cardio-vascular surgery and due to hypovolemia after hemodialysis [1-3]. Furthermore NOMI is a rare adverse event of medication with beta-blockers or digitalis [4,5]. The incidence of NOMI increases with age [2]. NOMI is about five times less common than acute bowel infarction caused by vascular occlusion of the superior or inferior mesenteric artery with its distinct morphological patterns [6-8].

The predilection site of NOMI is the right-sided colon [1]. However, as arteriosclerotic mesenteric disease is found very often in patients at risk for NOMI and the results of imaging and the intraoperative findings regarding occlusion of branches of the superior mesenteric artery are often equivocal, it is difficult to distinguish NOMI from bowel infarction caused by vascular occlusion.

Other entities with bowel infarction to be ruled out are ischemic colitis (rarely requiring surgical therapy, without predilection site) [9], venous mesenteric infarction [10], infarction due to strangulation ileus [11] or bowel ischemia due to local compression [12]. However, these entities are diagnosed by histological examination of the resected specimen.

Early diagnosis of NOMI remains a challenge as symptoms are often non-specific [13]. If acute mesenteric ischemia is suspected, the diagnostic method of choice is multi-detector row computed tomography with intravascular contrast enhancement with the possibility to detect alterations of the colonic wall [7]. Diagnostic laparoscopy could be a valuable option, although the estimation of bowel viability by means of laparoscopy is limited [14]. Some authors recommend conventional intra-arterial angiography with its possibility of intervention [15]. If NOMI is diagnosed early, a conservative treatment with prostaglandin infusion is an option [15,16]. However, bowel resection is often required as bowel infarction is already present in most cases at the time NOMI is diagnosed [13]. Primary anastomosis is only recommended if systemic conditions have improved [16] and most cases need an ileostomy without primary anastomosis. As an option, some authors recommend primary anastomosis with planned second look in patients with acute right-sided colonic ischemia in absence of peritonitis [14]. In small case series this planned second look has been successfully done laparoscopically [17], but as mentioned above surgeons have to consider that the estimation of bowel viability by means of laparoscopy is limited [14]. Another possibility could be damage control surgery with abdominal vacuum and delayed bowel reconstruction as proposed for perforated diverticulitis Hinchey III/IV [18].

However, due to low incidence of NOMI the knowledge about this disease is only based on small case series [19]. Furthermore little is known about the optimal surgical treatment and outcome of patients with this rare but life-threatening disease.

As it was not possible to unequivocally distinguish NOMI from mesenterial infarction caused by occlusion of branches of the superior mesenteric artery, we assessed the short and long-term outcome and especially the rate of ostomy reversal in patients with acute right-sided colonic ischemia without feasible vascular intervention as a surrogate for NOMI.

## Methods

Between January 2007 and January 2013 a total of 70 patients were identified in the institution’s prospectively maintained colorectal database, who had urgent surgery for acute right-sided colonic ischemia without feasible vascular intervention as judged by a senior interventional radiologist.

Patients with exploration only, with right-hemicolectomy or (sub-)total colectomy and histopathological confirmation of ischemia were included. Patients with missing histology despite resection (n = 2), toxic megacolon and/or pure hemorrhagic infarction (n = 9), and amyloidosis (n = 1) were excluded.

Thus a total of 58 patients remained for analysis. These were classified into the following groups according to the surgical treatment: (extended) right-sided hemicolectomy with primary anastomosis; (extended) right-sided hemicolectomy with creation of an ostomy; (sub-) total colectomy with ostomy creation; exploration only.

**Figure 1:**
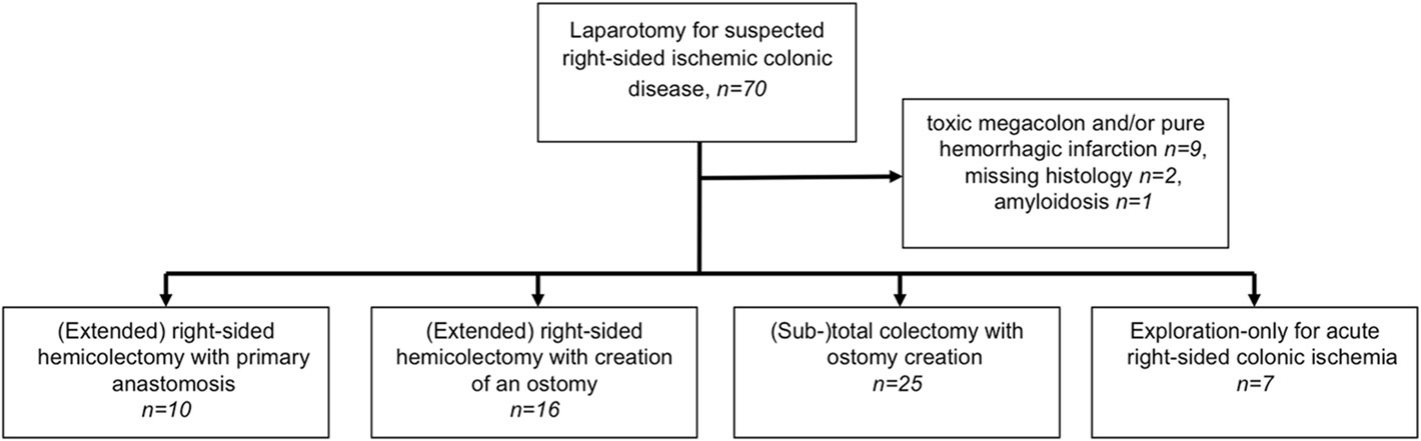
**An overview of the study methodology.**

An overview of the study methodology is shown in Figure 1.

Patients’ characteristics, reason for ischemia, extent of resection, creation of ostomy versus primary anastomosis, 30 day mortality, duration of hospital stay of the patients surviving 30-days, rate of ostomy reversal, and 1 year mortality were assessed.

Statistics: Results are expressed as median and range. Pearson’s chi square test was used for categorical data. The Kruskal-Wallis test was used for continuous variables. Significance was set at the p < 0.05 level (two-sided).

### Ethics statement

The present study is a retrospective observational study without any potential harm for patients. It is based on a prospectively maintained colorectal database. A statement by the responsible ethics committee allows the study group to maintain this prospectively maintained database on patients for scientific research (Ethikkommission der Fakultät für Medizin der Technischen Universität München, Nr. 1926/07).

## Results

Ten patients had (extended) right-sided hemicolectomy with primary anastomosis, sixteen patients had (extended) right-sided hemicolectomy with creation of an ostomy. Twenty-five patients had (sub-) total colectomy with ostomy creation, and seven patients had exploration only due to infaust prognosis. No patient with (sub-) total colectomy had primary anastomosis done.

The groups are comparable regarding the patients’ characteristics (Table 1). The only significant difference between the groups was American Society of Anesthesiologists (ASA) score, with the worst score for the group of patients with exploration only. Of all patients who had a preoperative CT scan (n = 39), 21 patients (54%) had arteriosclerotic mesenteric disease but only n = 6 (15%) had suspected concomitant vaso-occlusive disease. However, during surgery the vaso-occlusive disease could not be confirmed.

**Table 1:** **Patients’ characteristics**

	**Total**	**(Extended) right-sided hemicolectomy with primary anastomosis**	**(Extended) right-sided hemicolectomy with creation of an ostomy**	**(Sub-)total colectomy with ostomy creation**	**Exploration only (30d mortality 86%)**	**p-value (comparing all fields)**
**Number of patients**	58	10	16	25	7	
**Male gender**	30	5	8	13	4	p = 0,990
**Age: Median (Range)**	70,3 years (31,7-95,8)	70,7 years (31,7-87,5)	70,6 years (51,3-86,6)	70,4 years (56,8-92,3)	61,7 years (60,8-83,7)	p = 0.786
**Cardiovascular co-morbidities**	45	8	11	21	5	p = 0,683
**Suspected occlusion of the superior mesenteric artery or its branches**	6/39	2/8	2/11	1/16	1/4	p = 0,584
**Atherosclerotic mesenteric arterial disease**	21/39	4/8	9/11	5/16	3/4	p = 0,204
**Acute cardiac event prior to surgery**	15	3	1	10	1	p = 0,094
**Renal comorbidities**	17	2	4	8	3	p = 0,736
**Pulmonary comorbidities**	13	1	3	6	3	p = 0,435
**Hepatic comorbidities**	7	1	2	3	1	p = 0,993
**Neurological comorbidities**	7	2	1	3	1	p = 0,768
**Diabetes mellitus**	8	2	0	5	1	p = 0,296
**ASA-score (mean)**	3.4	2.6	3.6	3.3	3.7	p = 0.010

ASA: American Society of Anesthesiologists.

No anastomotic leak occurred in the group of patients with primary anastomosis. The 30-days mortality rate of all patients together was 52%. An overview of the significantly different 30-day mortality rates of the different groups is shown in Table 2. The highest 30-day mortality rate was in the group with exploration only. The duration of hospital stay of the patients surviving 30 days (median 22 days) is also shown in Table 2. Two patients were lost to follow-up after discharge from hospital. The one-year mortality-rate of all patients together was 70%. The one-year mortality-rates were significantly different between the groups (Table 2). Ostomy reversal was done in three patients (7 % of all patients with ostomy, but in 16% of those patients with ostomy alive at 30-days postoperative (n = 19).

**Table 2:** **Mortality, hospital stay after surgery and ostomy reversal rates**

	**Total**	**(Extended) right-sided hemicolectomy with primary anastomosis**	**(Extended) right-sided hemicolectomy with creation of an ostomy**	**(Sub-)total colectomy with ostomy creation**	**Exploration only (30d mortality 86%)**	**p-value (comparing all fields)**
**Total number of patients**	58	10	16	25	7	
**30-day mortality**	30(52%)	2(20%)	7(44%)	15(60%)	6(86%)	p = 0,039
**Median (range) of hospital stay after surgery of those patients surviving 30 days**	22 days (5–220)	14.5 days (9–26)	32 days (13–96)	24 days (5–220)	40 days	p = 0,057
**1-year mortality**	39/56(70%)	3/10(30%)	12/14(86%)	18/25(72%)	6/7(86%)	p = 0.018
**Ostomy reversal**	n/A	n/A	1/9(11%)	2/10(20%)	n/A	p = 1.000

n/A: not applicable.

## Discussion

We aimed to assess the surgical therapy, short and long term outcome and the rate of ostomy reversal in patients with right-sided colonic ischemia without vascular intervention.

One challenge was to draw a line between patients with NOMI and those with questionable occlusion of the branches of the superior mesenteric artery. The radiological and intraoperative findings often are equivocal regarding presence of vaso-occlusive disease. Although, the typical morphologic pattern of bowel ischemia due to complete occlusion of the superior or inferior mesenteric artery is quite distinct to right sided colonic ischemia [1] and mixing them up seems unlikely, the obstruction of a branch of the superior mesenteric artery can mimic NOMI. This is why we defined “acute right-sided colonic ischemia without feasible vascular intervention” as a surrogate for NOMI.

In a six year period we identified 58 patients who underwent surgery for acute right-sided colonic ischemia without feasible vascular intervention. Less than 20% of the patients had colonic resection and primary anastomosis. As to be expected there were significant differences in the ASA scores between the groups, reflecting the extent of disease. As to be expected patients with lower ASA scores were more likely to receive a primary anastomosis. For all patients together the 30-days mortality was 52% and 1-year mortality was as high as 70% reflecting the hazardousness of this acute right-sided colonic ischemia. There were significant different mortality rates between the groups. However, compared with historical controls these mortality rates are lower than expected [3,20]. Overall less than 10% of the ostomies were reversed, and only about 15% of those patients surviving 30 days had ostomy-reversal.

The strength of this study is the comparatively high number of patients with this rare diagnosis if compared to hitherto reported series. However, due to the retrospective nature of this study there are several limitations.

Although there was no standardized follow-up, and most of the data was collected retrospectively, only two patients (3%) were lost to follow-up.

As no anastomotic leak occurred in the group of patients with primary anastomosis, it could be argued that more patients should have had primary anastomosis with planned second look laparotomy. However, with regard to the high 30 days mortality rate of 52%, primary anastomosis with planned second-look [14] seems to be no good option in this life threatening condition of patients with acute right-sided colonic ischemia. This is confirmed by anastomotic leakage rates of 33% reported from a comparable cohort of patients [20]. However damage control surgery with abdominal vacuum and delayed bowel reconstruction as proposed for perforated diverticulitis Hinchey III/IV [18] might be a valuable option.

To confirm our results, a prospective study with standardized pre-operative work-up is required. However, it would be a challenge to run such a study as NOMI has a very low incidence and patients with NOMI often present with nonspecific symptoms and signs. Last but not least there would be ethical concerns to perform such a study in patients with this life-threatening disease with a potentially disturbance of consciousness.

## Conclusion

Patients who need urgent surgery for acute right-sided colonic ischemia without feasible vascular intervention have a very high short and long-term mortality probably due to their multiple and severe underlying diseases. Less than 10% of the constructed ileostomies are reversed.

## Consent

The present is a true retrospective observational study. Single patients cannot be identified based on the data provided. Thus, there is no potential harm for patients. The 1-year mortality rate was 70%. Furthermore two patients were lost to follow-up. These are the reasons why written informed consent could not be obtained from the majority of the patients. However, we feel that from an ethical point of view, this should not hinder publication of the results of this study.
